# Profiling cellular morphodynamics by spatiotemporal spectrum decomposition

**DOI:** 10.1371/journal.pcbi.1006321

**Published:** 2018-08-02

**Authors:** Xiao Ma, Onur Dagliyan, Klaus M. Hahn, Gaudenz Danuser

**Affiliations:** 1 Dept. of Bioinformatics, University of Texas Southwestern Medical Center, Dallas, Texas, United States of America; 2 Department of Pharmacology, University of North Carolina at Chapel Hill, Chapel Hill, North Carolina, United States of America; Northeastern University, UNITED STATES

## Abstract

Cellular morphology and associated morphodynamics are widely used for qualitative and quantitative assessments of cell state. Here we implement a framework to profile cellular morphodynamics based on an adaptive decomposition of local cell boundary motion into instantaneous frequency spectra defined by the Hilbert-Huang transform (HHT). Our approach revealed that spontaneously migrating cells with approximately homogeneous molecular makeup show remarkably consistent instantaneous frequency distributions, though they have markedly heterogeneous mobility. Distinctions in cell edge motion between these cells are captured predominantly by differences in the magnitude of the frequencies. We found that acute photo-inhibition of Vav2 guanine exchange factor, an activator of the Rho family of signaling proteins coordinating cell motility, produces significant shifts in the frequency distribution, but does not affect frequency magnitude. We therefore concluded that the frequency spectrum encodes the wiring of the molecular circuitry that regulates cell boundary movements, whereas the magnitude captures the activation level of the circuitry. We also used HHT spectra as multi-scale spatiotemporal features in statistical region merging to identify subcellular regions of distinct motion behavior. In line with our conclusion that different HHT spectra relate to different signaling regimes, we found that subcellular regions with different morphodynamics indeed exhibit distinct Rac1 activities. This algorithm thus can serve as an accurate and sensitive classifier of cellular morphodynamics to pinpoint spatial and temporal boundaries between signaling regimes.

## Author summary

Many studies in cell biology employ global shape descriptors to probe mechanisms of cell morphogenesis. Here, we implement a framework in this paper to profile cellular morphodynamics very locally. We employ the Hilbert-Huang transform (HHT) to extract along the entire cell edge spectra of instantaneous edge motion frequency and magnitude and use them to classify overall cell behavior as well as subcellular edge sectors of distinct dynamics. We find in fibroblast-like COS7 cells that the marked heterogeneity in mobility of an unstimulated population is fully captured by differences in the magnitude spectra, while the frequency spectra are conserved between cells. Using optogenetics to acutely inhibit morphogenetic signaling pathways we find that these molecular shifts are reflected by changes in the frequency spectra but not in the magnitude spectra. After clustering cell edge sectors with distinct morphodynamics we observe in cells expressing a Rac1 activity biosensor that the sectors with different frequency spectra associate with different signaling intensity and dynamics. Together, these observations let us conclude that the frequency spectrum encodes the wiring of the molecular circuitry that regulates edge movements, whereas the magnitude captures the activation level of the circuitry.

## Introduction

Cell morphology and morphodynamics are used to phenotype the state of a cell throughout various processes, including differentiation, proliferation, migration and apoptosis[1–5]. Moreover, numerous signaling pathways converge onto cytoskeleton architecture that determines morphological variation among cells. Therefore, parameters of cell morphology and morphodynamics can also serve as indicators of signaling states[6, 7]. Indeed, analysis of cellular morphology and morphodynamics has been applied, for example, in cancer cell screens[8], drug development[9–11], cell transformation characterization[12] and cell cycle analysis[13, 14].

A number of strategies have been developed to elucidate the physical cause and signaling regulation of cell morphology. Quantification of cell edge movements using physical and mathematical models revealed different modes of motility associated with actin-based spreading[6, 15–25], myosin-related contraction[26] and transverse wave propagation[22, 27–31]. Moreover, shape descriptors have been used for statistical classification of cell morphological patterns[32–38]. However, these studies generally applied a global parameterization of cell morphological changes, such as degree of polarization, cell area change and migration rate, and did not consider the local and dynamic behavior of the cell edge. This has been in part due to the significant complexities in robustly tracking cell edge motion at the subcellular scale. We[39] and others[40] have developed the necessary image analysis framework to track complex cell boundary movements in time-lapse cell image sequences. Densely sampled protrusion and retraction velocities were compiled in space-time maps that offer an opportunity to identify distinct cell morphodynamic states as well as to unveil putative functional links to underlying cytoskeleton dynamics[41–44] and signaling activities[45–47]. Nonetheless, a systematic classification of the spatiotemporal patterns captured by these maps has yet to be performed.

Here, we implement a framework based on the Hilbert-Huang Transform (HHT) to decompose the spatiotemporal signal into instantaneous frequencies and amplitudes. Applied to a population of spontaneously migrating fibroblast-like Cos7 cells, we show that the frequencies encode information on the wiring topology of pathways involved in the regulation of morphodynamics, whereas the amplitudes reflect pathway activation levels. We then validate these results by acute manipulation of the wiring topology of a pathway using optogenetics[38]. We also show that the decomposition into temporally and spatially localized frequency spectra offers an opportunity to identify time windows and cell edge sectors with distinct morphodynamic signatures. This permits determination with subcellular resolution of switches between morphodynamic states that are associated with particular signaling motifs.

## Results

### Spatiotemporal sampling of local cell edge movement

**Fig 1:**
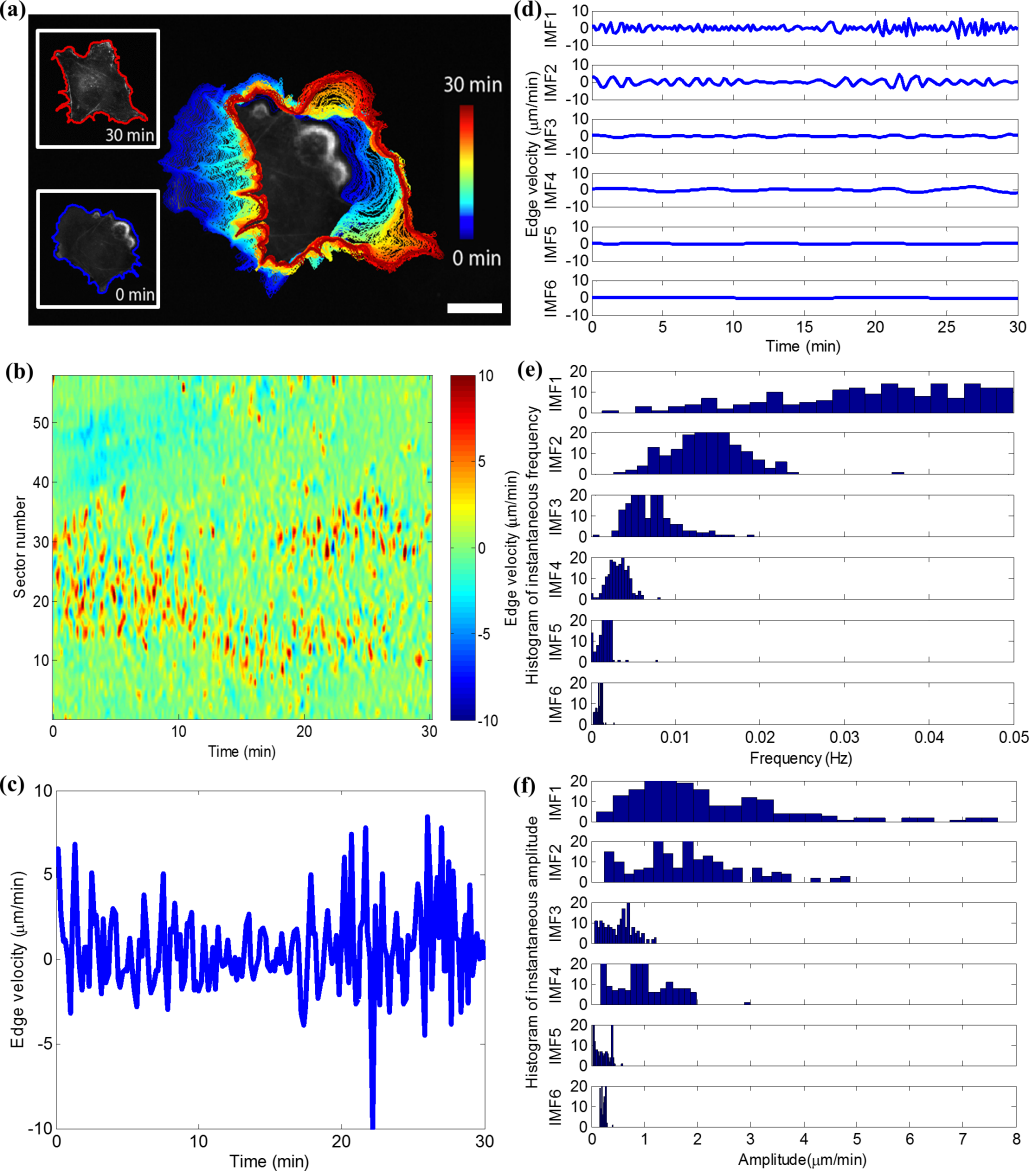
Spectral decomposition of cell edge velocity time series. (a) A Cos7 fibroblast-like cell with overlaid cell boundaries sampled at 10 sec intervals for 30 min, showing early (blue) and late (red) time points; scale bar = 20 μm. (b) Protrusion activity map displaying the dynamics of all sectors along the cell edge. (c) Edge velocity time series representing the randomly oscillating motion of a particular edge sector (sector No. 30). (d) Empirical mode decomposition (EMD) of time series in (c). (e) Instantaneous frequency distributions of each intrinsic mode function (IMF) calculated by the Hilbert transform. (f) Instantaneous amplitude distributions of each IMF calculated by the Hilbert transform.

We hypothesized that subcellular morphodynamic profiling would be highly informative regarding the states of signaling pathways that regulate cytoskeleton and adhesion dynamics at the cell periphery. To test this, we first imaged unstimulated Cos7 monkey kidney fibroblast-like cells. These cells often exhibit a robust spontaneous migration, and because of their tight adhesion to the substrate, are ideal for high-resolution live cell imaging. We tracked the motion of virtual fiduciaries on the cell boundary by identifying the outline of the cell edge in each frame of a time-lapse sequence, and mapping the outlines of consecutive frames subject to minimizing the overall displacement and strain that are associated with the deforming cell shape[39, 48] (Fig 1A, see Methods and S1 Fig for details on the mapping strategy). We subsequently sampled time series of local protrusion (positive velocities) and retraction (negative velocities) by averaging the motion within edge sectors of ~10 pixel (i.e. ~3 μm) width each. This spacing in sampling corresponds to the half-width-at-half-maximum (HWHM) of the spatial autocorrelation of the edge motion[49]. Velocity time series along the cell boundary were then compiled sector-by-sector into the rows of a matrix referred to as a *protrusion activity map*[39] (Fig 1B). Accordingly, a matrix column represents the velocity variation over all edge sectors in a particular time point. For the particular cell displayed in Fig 1A, the boundary region encompassing sectors 12–38 prominently protrudes for the first 15 min of the movie, interspersed with short periods of retraction. After 15 min the region splits into two protrusive subregions. The boundary region encompassing sectors 40–54 retracts for the first 10 min before converting into a relatively quiescent zone (see also Video 1). These examples show that the velocity time series is nonstationary. Accordingly, edge motion analysis must be temporally localized.

### Spectral analysis of velocity time series using Hilbert–Huang transform

To analyze edge velocity time series, we adopted the Hilbert–Huang transform (HHT)[50–55]. The HHT relies on an empirical mode decomposition (EMD), which divides the submitted time series into a finite and generally small number of component signals, referred to as *intrinsic mode functions* (IMFs). The set of IMFs forms a complete and nearly orthogonal basis for the original signal satisfying the following two conditions: i) The number of local extrema and the number of zero-crossings either is equal to each other or at most differs by one. ii) The mean value of the upper envelope defined by the local maxima and the lower envelope defined by the local minima is equal to zero. Under these conditions, the Hilbert Transform is guaranteed to converge to an unbiased estimate of the instantaneous frequency spectrum of the IMF[50, 54].

The EMD procedure involves iterative application of the following steps: i) Identifying all local extrema in the original target time series *X*(*t*). ii) Connecting all local maxima by a cubic spline to generate the upper envelope; similarly, connecting all local minima by a cubic spline to generate the lower envelope. iii) Computing the mean *m*_1_(*t*) of upper and lower envelopes and subtracting it from the target time series to generate a reduced series *h*_1_(*t*).


$$X(t)-m_{1}(t)=h_{1}(t)$$

If *h*_1_(*t*) satisfies the aforementioned conditions of an IMF, it is the first IMF component *c*_1_(*t*). Usually this is not the case. Instead *h*_1(1)_(*t*) = *h*_1_(*t*) is considered the new target time series, and the above procedure is repeated *k*-1 times,

$$\begin{array}{c} h_{1(1)}(t)-m_{1(2)}(t)=h_{1(2)}(t)\\ ⁞\\ h_{1(k-1)}(t)-m_{1(k)}(t)=h_{1(k)}(t) \end{array}$$

until *h*_1(*k*)_(*t*) satisfies the conditions of an IMF. This is the first IMF component *c*_1_(*t*).


$$c_{1}(t)=h_{1(k)}(t)$$

The residual signal *r*_1_(*t*) is then defined as

$$r_{1}(t)=X(t)-c_{1}(t)$$

and used in a next iteration as the initial target time series. Usually, the decomposition is terminated after *n* iterations, subject to the condition that the residual signal is either a constant, or a monotonic function, or a function with only one maximum and one minimum, from which no more IMF can be generated. However, in our application IMF sets were compared between experiments. Therefore, it was necessary to fix the number of iterations such that the majority of decomposed data fulfilled the above defined termination criterion. Irrespective of the termination rule, the EMD generates *n* IMF components *c*_1_(*t*), …, *c*_*n*_(*t*) and a residual signal *r*_*n*_(*t*) that satisfy

$$X(t)=\sum_{i=1}^{n}c_{i}(t)+r_{n}(t)$$

where *r*_*n*_(*t*) either fulfills the above termination criterion or its variance is less than, e.g., 5% of that of the original target time series *X*(*t*).

Application of the Hilbert Transform to a particular IMF produces an instantaneous frequency spectrum at each time point *t*.

$$H[c_{i}(t)]=\frac{1}{\pi }\int_{-\infty }^{\infty }\frac{c_{i}(\tau )}{t-\tau }d\tau$$ (1)

$$F(t)=\frac{1}{2\pi }\cdot \frac{d}{dt}\left(\arctan\left(\frac{H[c_{i}(t)]}{c_{i}(t)}\right)\right)$$ (2)

$$A(t)=\sqrt{c_{i}{}^{2}(t)+H^{2}[c_{i}(t)]}$$ (3)

where *i* = 1, …, *n*. The instantaneous frequency spectrum is the temporal derivative of the phase change in the IMF signal *c*_i_(*t*), which is defined by the inverse tangent function of the quotient between the Hilbert Transform of the original signal *c*_i_(*t*) (see Eq (1)) and the original signal *c*_i_(*t*) (see Eq (2)). The corresponding instantaneous amplitude spectrum is the root of the square sum of the original signal *c*_i_(*t*) and its Hilbert Transform (see Eq (3)).

We show an example of the decomposition of the velocity time series at a specific sector (Fig 1C) in Fig 1D. By definition of the EMD procedure, higher order IMFs (Fig 1D) tend to contain lower frequencies and lower amplitudes. However, in our data the instantaneous frequency and amplitude spectra at a specific sector overlapped between IMFs (Fig 1E and 1F). We computed the frequency and amplitude spectra for all IMFs in all edge sectors, which generated at each time point for each cell boundary sector six temporal frequency and amplitude values. Moreover, we repeated the HHT computation for all columns of the protrusion activity map to capture the instantaneous spatial frequency and amplitude spectra. As with the time domain, we restricted the EMD to six spatial IMFs, which generated at each cell boundary sector for each time point another six spatial frequency and amplitude values. We chose the number (six) of IMFs empirically and found it works well to capture the variation of complex cellular morphodynamics.

To illustrate the meaning of the EMD and to better interpret the related spectral decomposition outcomes, we reconstructed six movies and associated activity maps that visualize the cell edge motion captured by the six IMFs (Fig 1D, S2 Fig and and Video 2). For a particular IMF at a particular cell edge sector we extracted time point by time point the velocity magnitude and integrated the values into a displacement time series (Fig 1C). After computing the displacements for all sectors in one time point we plotted the virtual cell edge and repeated the procedure for all time points to generate a movie associated with the IMF. Each of the six movies starts with the true cell edge image at the first time point. Video 2 clearly indicates the distinct levels of motion persistence and magnitude captured by the six IMF signals. For example, IMF1 captured the protrusion signal with highest frequency and greatest magnitude, which yields rapid and jerky changes in cell shape. In contrast, IMF6 captured only subtle long-range position changes of the cell edge with almost no shape change associated. Hence, the instantaneous frequencies extracted from these different IMF orders represent, on average, different length scales and ranges of persistence in the protrusion-retraction cycles of a cell.

### Frequency distribution is conserved for spontaneously protruding cells

**Fig 2:**
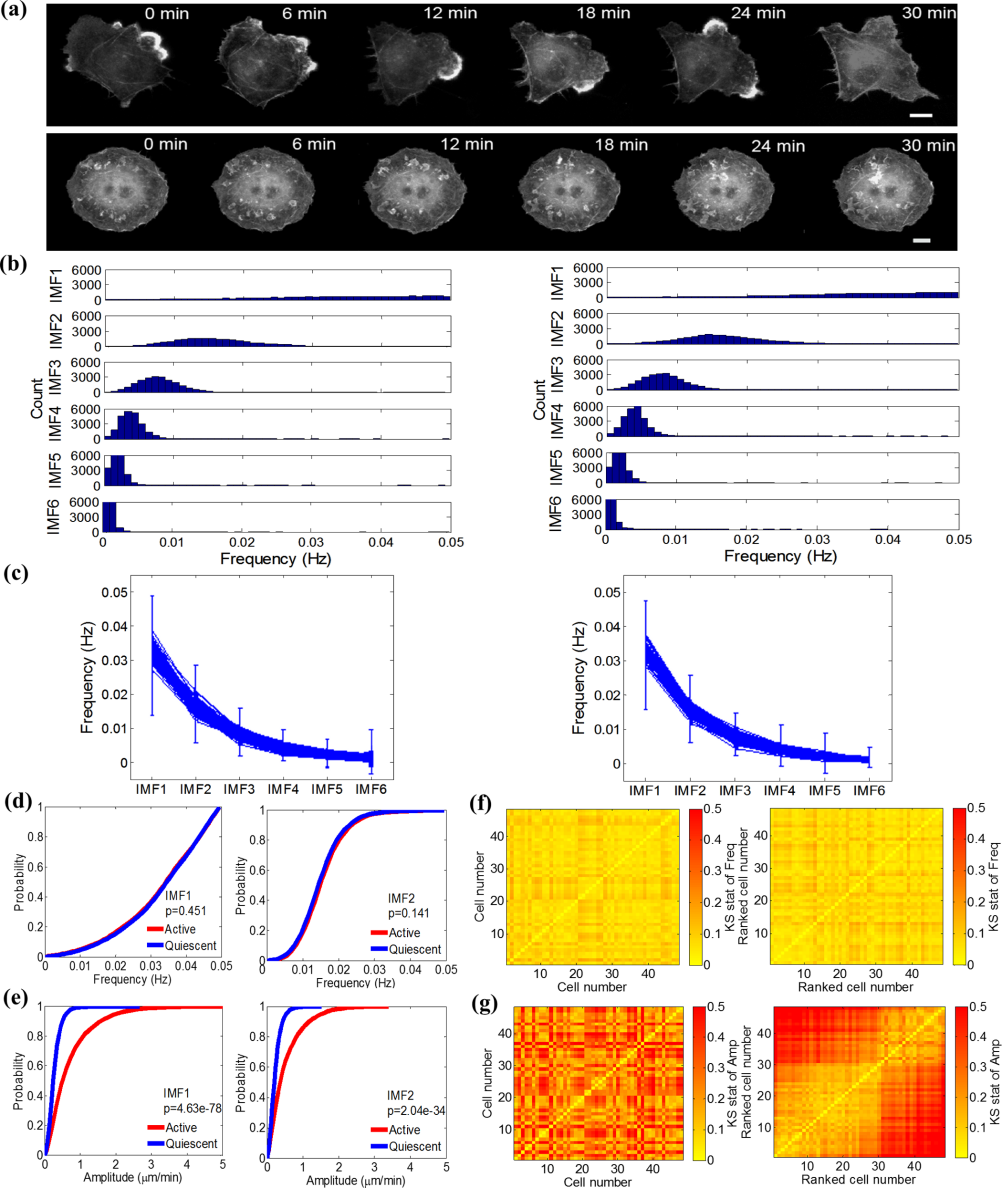
Conservation of frequency distribution among a population of spontaneously protruding Cos7 cells. (a) Snapshots of active and quiescent Cos7 cell protrusion for 30 min with an increment of 6 min, scale bar = 20μm (see also Video 1 and 3). (b) Histograms of instantaneous frequency distribution for active vs quiescent Cos7 cell. (c) Exponential decrease of instantaneous frequency distributions captured by IMF1-6 for active and quiescent Cos7 cell. Each curve corresponds to the central frequencies of six IMFs computed from a specific sector. The error bar of each IMF indicates the standard error of the instantaneous frequency distribution over all sectors. (d) Cumulative distribution function (CDF) comparison of instantaneous frequency distributions for IMF1 and IMF2 between an active and a quiescent Cos7 cell. P-value is calculated by Kolmogorov–Smirnov (K-S) test. (e) CDF comparison of instantaneous amplitude distribution for IMF1 and IMF2 between an active and a quiescent Cos7 cell. P-value is calculated by K-S test. (f) Heatmaps of KS-statistics for instantaneous frequency distributions among 48 spontaneously protruding Cos7 cells. The sequence of cells is defined in randomized (left) and ascending order (right) of the mean absolute velocity over all cell edge sectors and time points. (g) Heatmaps of KS-statistics for instantaneous amplitude distributions among 48 spontaneously protruding Cos7 cells. The sequences of cells are defined in the same orders as in panel (f).

We first applied the spectral decomposition to the edge movements of spontaneously protruding Cos7 fibroblasts. These cells exhibited a wide range of cell shapes and morphodynamics at a basal level of activity. For example, some cells showed persistent polarity and protruded/retracted over large parts of their peripheries (top panel in Fig 2A and Video1). Other cells showed an unpolarized morphology with only small oscillatory edge movements along the entire periphery (lower panel in Fig 2A and Video 3). For the two cells illustrated in Fig 2A, we extracted histograms of instantaneous frequencies from each of the six IMFs (Fig 2B). Despite the vast differences in cell shape and motion, the two sets of histograms appeared strikingly similar. For both active and quiescent cell, the central frequencies of IMFs decreased exponentially (Fig 2C). Comparison of cumulative distribution functions (CDFs) using Kolmogorov–Smirnov (K-S) test statistics confirmed that the frequency spectra of the two cells were statistically indistinguishable (Fig 2D and S3 Fig). In contrast, the K-S test statistics of the instantaneous amplitudes were different (Fig 2E and S3 Fig). This observation also held for 48 spontaneously protruding Cos7 cells (Fig 2F and 2G). The instantaneous frequency distributions of cells with comparable molecular makeup and similar levels of stimulation were conserved regardless of morphological and morphodynamic differences. In contrast, morphological and morphodynamic differences manifested themselves in significant variations of the amplitude spectra. The more different the velocities of two cells were, the larger the difference between their instantaneous amplitude spectra (Fig 2G)). Of note, the small differences between instantaneous frequency spectra were independent of the cell order (Fig 2F). Those analyses indicate the orthogonality between instantaneous amplitude and frequency spectra in capturing cell morphodynamic behaviors.

### Photoinhibition of Vav2 signaling leads to shift in instantaneous frequency

**Fig 3:**
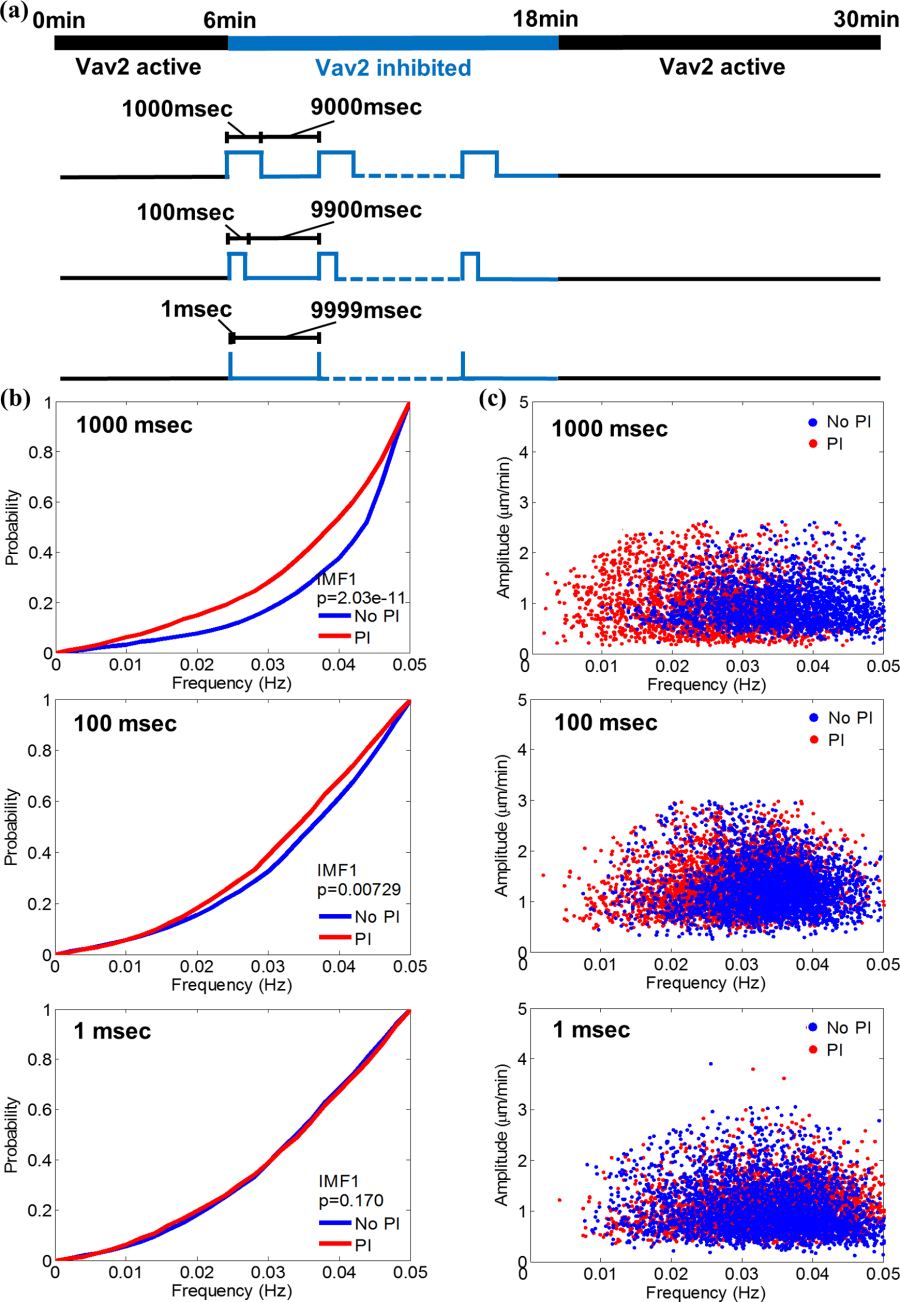
Acute photo-inhibition (PI) of Vav2 signaling induces a shift in the instantaneous frequency distribution. (a) Timing of Rac-GEF Vav2 photo-inhibition using PI Vav2. (b) Comparison of IMF1 instantaneous frequency distributions collected before and during a PI period composed of 1000 msec pulses of light interspersed with 9000 msec darkness, 100 msec pulses of light interspersed with 9900 msec darkness, and 1 msec pulses of light interspersed with 9999 msec darkness (from top to bottom). P-value is calculated by K-S test. Data for IMF2-6 are presented in S4 Fig. (c) Representative scatter plots of instantaneous frequency vs amplitude values of IMF1 before and during a PI period induced by 1000, 100 and 1 msec of light pulses (from top to bottom), respectively.

The conservation of instantaneous frequency distribution in molecularly similar, spontaneously migrating Cos7 cells led us to ask whether induced shifts in morphogenetic signaling greater than the basal level of variation in a control cell population would systematically alter the frequency components. To address this, we employed a recently introduced optogenetic construct that allows acute and reversible inhibition of the guanosine exchange factor (GEF) Vav2[38]. We have previously shown that Vav2 acts as a core element of a signaling resonator that controls the oscillatory protrusion and retraction of cells[56]. To capture the morphodynamic response to Vav2 inhibition, we filmed cells for 6 minutes without light-activation of the inhibitor construct, followed by 12 min of pulsed blue-light inhibition, and then another 12 min in the dark to examine the recovery of Vav2 activation levels (Fig 3A). Activation pulses of three different lengths were examined: 1000 msec, 100 msec, and 1 msec (Fig 3A).

Based on the comparison of instantaneous frequency distributions, photo-inhibition with pulse lengths of 1000 msec and 100 msec changed the spectra. We did not observe any evident change with a pulse length of 1 msec (see Fig 3B). Importantly, the shifts were limited to the first three IMFs, which covered frequencies in a range 0.006–0.035 Hz (S4 Fig). Frequencies below this band were unaffected. Overall, inhibition of Vav2 signaling yielded lower frequencies, suggesting that this signal is implicated in pathways that promote fast exploratory protrusion and retraction cycles. Strikingly, scatter plots of frequency versus amplitude indicate that Vav2 inhibition has no effect on the amplitude (Fig 3C), *i*.*e*. the speed of the protrusion-retraction cycles. Those experiments suggest a separation of pathways that set the pace of the protrusion machinery from pathways that define the power of this same machinery.

### Definition of cell edge motion regimens by spectral feature region merging

**Fig 4:**
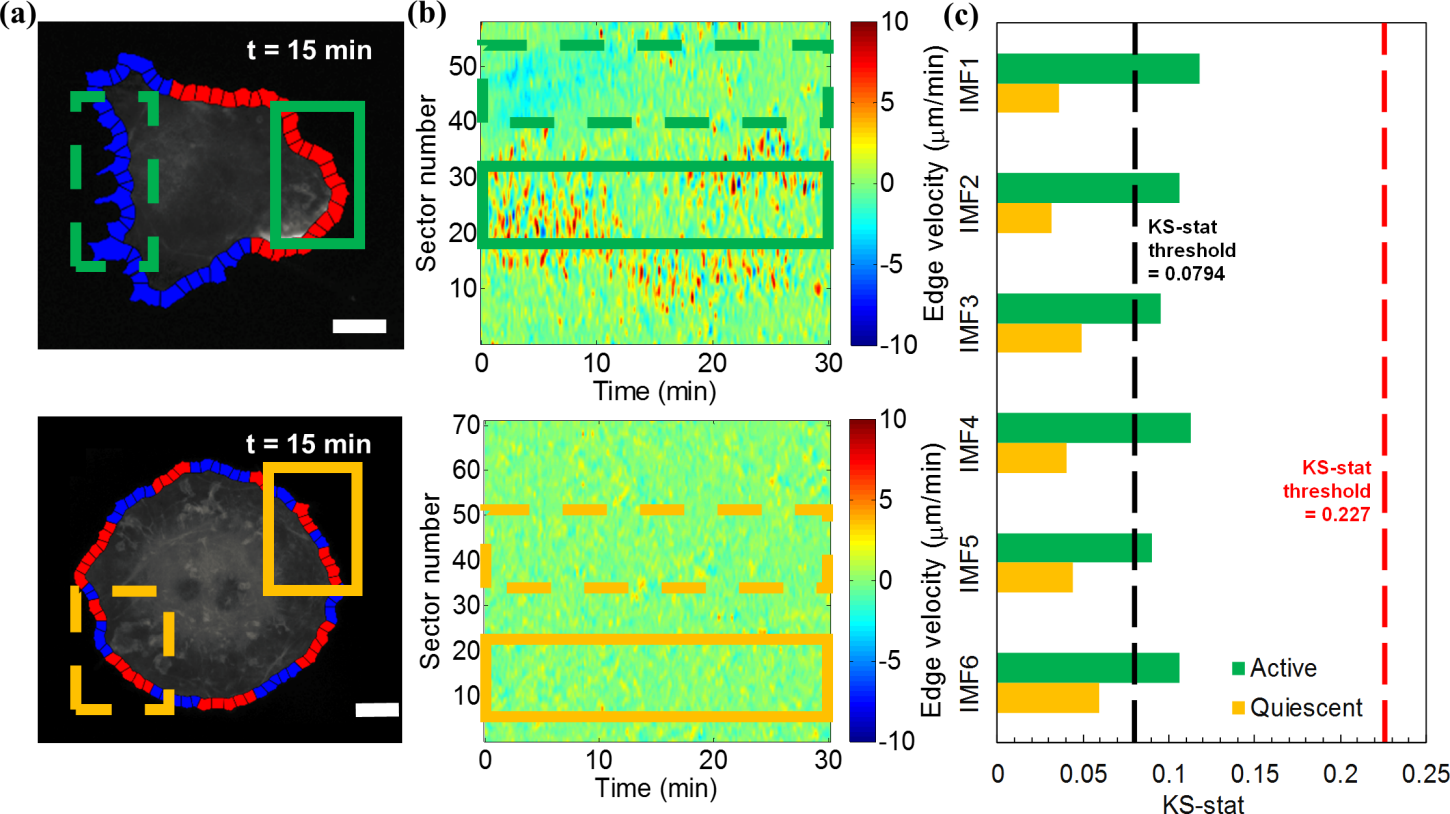
Polarity of spontaneously protruding Cos7 cells results in different spectral responses of subcellular regions. (a) Snapshots of Cos7 cell protrusion at 15 min with red colored windows corresponding to higher motility (≥ 2μm/min) and blue colored windows corresponding to lower motility (< 2μm/min).See also Video 4 and 5. Scale bar = 20μm. Top: active, polarized cell; Bottom: quiescent cell. The solid green box in upper panel indicates the selected protruding cell front, and the dashed green box indicates the selected quiescent cell rear. The solid and dashed orange boxes in lower panel indicate the two subcellular regions of the quiescent Cos7 cell randomly selected for comparison. (b) Protrusion activity maps for active (top) and quiescent (bottom) cells. The solid/dashed green and yellow boxes indicate the time series selected for spectral analysis. (c) K-S test statistics comparing the instantaneous frequency distributions between the indicated subcellular regions. The threshold K-S statistics value of 0.0794 (black dash line) indicates the average difference between whole-cell distributions for a population of cells with homogeneous molecular make-up (mean value of K-S statistics heatmap Fig 2F). The threshold value of 0.227 (red dash line) indicates the average difference between whole-cell distributions of control and Vav2-inhibited cells.

In the experiments described thus far, we used instantaneous frequency and amplitude as morphodynamic signatures reflecting the state of an entire cell with sufficient sensitivity. We then asked if those signatures could also be applied to distinguish the potentially transient signaling states of subcellular regions. We first evaluated the spectral signatures of a migrating Cos7 cell with obvious polarity (Fig 4A and 4B, upper panels). The subcellular region indicated by the solid green box represents the actively protruding cell front, whereas the region indicated by dashed green box represents the retracting/quiescent cell rear. We separately applied the HHT to the time series encompassed by these two regions, extracted the instantaneous frequency distributions and conducted the K-S test to obtain K-S statistics of all six IMFs. For comparison, we repeated this analysis on two randomly selected subcellular regions of a quiescent Cos7 cell (Fig 4A and 4B, lower panels). For all IMFs, the K-S statistics comparing the front to back dynamics in a polarized cell was greater than the K-S statistics comparing two randomly selected regions of a quiescent cell (Fig 4C). The former K-S statistics also systematically exceeded the average K-S statistics quantifying cell-to-cell variability in the population of control cells (Fig 4C, black dash line) analyzed in Fig 2. However, they did not exceed the level of K-S statistics that were related to the morphodynamic shifts induced by acute Vav2 inhibition (Fig 4C, red dash line). This suggests that the signaling changes we experimentally introduced were stronger than the differences in signaling programs between front and back of a polarized cell.

To further test the postulation that spectral signatures of cell edge motion could distinguish the signaling states of subcellular regions, we compiled the instantaneous temporal and spatial frequency and amplitude spectra in a feature vector at each time point and each location and performed statistical region merging (SRM)[57] to identify regions of the cell edge with distinct motion regimens. Specifically, we formulated two 12-dimensional vectors in each sector *s* at each time point *t* (Eq (4)). The vector contains components 1 to 6 of the instantaneous temporal frequencies at time point *t* computed from the sector’s six IMFs along the time axis. Components 7 to 12 contain the instantaneous spatial frequencies in sector *s* computed from the six IMFs capturing the cell edge undulations at time point *t* along the space axis. The vector represented in Eq (5) captures the instantaneous amplitudes in the same fashion.


$$F(s,t)={\left[F_{1,t}(s),\cdots ,F_{6,t}(s),F_{1,s}(t),\cdots ,F_{6,s}(t)\right]}^{T}$$ (4)

$$A(s,t)={\left[A_{1,t}(s),\cdots ,A_{6,t}(s),A_{1,s}(t),\cdots ,A_{6,s}(t)\right]}^{T}$$ (5)

$$\phi (s,t)=\left[F_{1,t}(s)\times \frac{A_{1,t}^{2}(s)}{A_{\max,t}^{2}(s)},\ldots \;,F_{6,t}(s)\times \frac{A_{6,t}^{2}(s)}{A_{\max,t}^{2}(s)},F_{1,s}(t)\times \frac{A_{1,s}^{2}(t)}{A_{\max,s}^{2}(t)},\ldots \;,F_{6,s}(t)\times \frac{A_{6,s}^{2}(t)}{A_{\max,s}^{2}(t)}\right]$$ (6)

The feature vector *ϕ(s*,*t)* in each sector *s* at each time point *t* is then composed of amplitude-weighted instantaneous temporal and spatial frequencies (Eq (6)). The amplitude weights are normalized by *A*_max,*t*_(*s*), which denotes the maximum amplitude for a specific sector along the time axis and by *A*_max,*s*_(*t*), denoting the maximum amplitude at a specific time point along the spatial axis. We chose quadratic amplitudes because they reflect the instantaneous relative energy consumed by a particular IMF in the temporal and spatial domain. In summary, the feature vector captures the instantaneous spectral properties that characterize the local morphodynamic activity of a particular sector at a particular time point.

We exploited the feature vector to identify in the protrusion activity map regions of homogeneous morphodynamics, i.e. regions of the cell edge that move over a specific time period under the same regimen. To define such regions we applied the SRM algorithm[57]. For a multi-dimensional feature vector, this algorithm merges two regions *R*_*1*_ *and R*_*2*_ if the difference in every feature component between the two regions is less than a threshold (Eq (7)). The threshold penalizes regions of very large area and includes a user-controlled merging delicacy parameter Q (Eq (8)). |*R*_*j*_| denotes the size of a region, and |*R*_*j*_|_max_ is an estimate for the largest region clustered in the map. Throughout this work, we set the value of |*R*_*j*_|_max_ to 256. *N*_*t*_ is the number of time frames in the cell imaging, and *N*_*s*_ is the number of sectors or windows along the cell periphery.


$$M(R_{1},R_{2})=\left\{\begin{array}{l} Merge\left|\phi_{i}(R_{1})-\phi_{i}(R_{2})\right|\leq \sqrt{T^{2}(R_{1})+T^{2}(R_{2})},\;\;\forall i=1,2\ldots 12\\ Separate\left|\phi_{i}(R_{1})-\phi_{i}(R_{2})\right|>\sqrt{T^{2}(R_{1})+T^{2}(R_{2})},\;\;\exists i=1,2\ldots 12 \end{array}\right\}$$ (7)

$$T(R_{j})=\sqrt{\frac{{\left|R_{j}\right|}_{\max}^{2}2^{-\left(Q\text{+}1\right)}}{\left|R_{j}\right|}\ln\left(\frac{{\left(\left|R_{j}\right|+1\right)}^{\min\left(\left|R_{j}\right|,\;{\left|R_{j}\right|}_{\max}\right)}}{1/\left(6N_{t}N_{s}\right)}\right)},\;\;j=1,2$$ (8)

**Fig 5:**
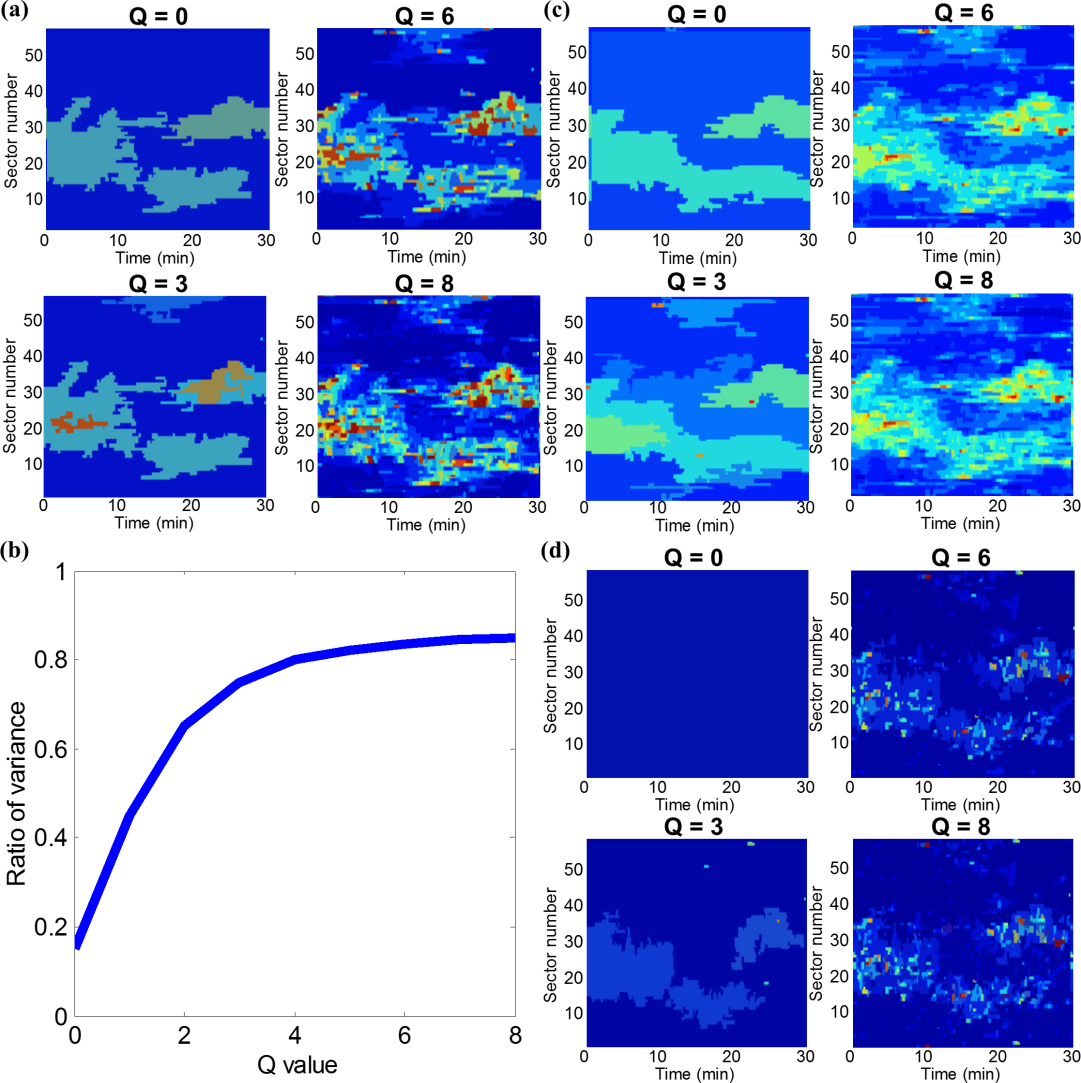
Spatiotemporal clustering of cell edge motion into spectrally homogeneous regions. (a) Statistical region merging (SRM) clustering at different Q-values (defining the delicacy of clusters) for a spontaneously protruding Cos7 cell using weighted instantaneous frequencies of all IMFs as a feature vector. (b) Ratio between intra- and inter-region variance as a function of the logarithm of Q. (c) Multiscale clustering using instantaneous amplitudes of all IMFs as a feature vector. (d) Multiscale clustering using weighted instantaneous frequency of only IMF1 as a feature vector.

The merging started with the feature vectors in individual edge sectors and time points and iteratively grew regions with sufficient similarity in morphodynamics until none of two regions in the protrusion activity map fulfilled the merging criteria. We showed the response of SRM to different levels of merging delicacy in Fig 5A. At Q = 0, only two protrusion regimens were differentiated, while at Q = 8 the activity map was decomposed into a high number of regimens that spanned very few sectors and lasted for only a few time points. To determine an optimal value for Q, we computed the ratio of intra- vs inter-region variance as a function of Q (Fig 5B). Beyond Q = 3 the fraction of explained variance increased only marginally, indicating that this level of granularity captures the spectrum of relevant morphodynamic regimens.

To demonstrate how critical the combination of instantaneous frequency and amplitude is for the formulation of a distinguishing feature vector, we compared the SRM results of the full feature vector using the combined instantaneous frequencies and amplitudes (Fig 5A) versus the results from using the instantaneous amplitudes only (Fig 5C). We also computed SRM results using the weighted instantaneous frequency of IMF1 only (Fig 5D). It is evident that the combined frequency and amplitude features accounting for all IMFs captures much finer spatiotemporal patterns. Thus, this feature vector is effective and suitable for SRM clustering.

### Application of spectral feature region merging in optogenetically perturbed cells

**Fig 6:**
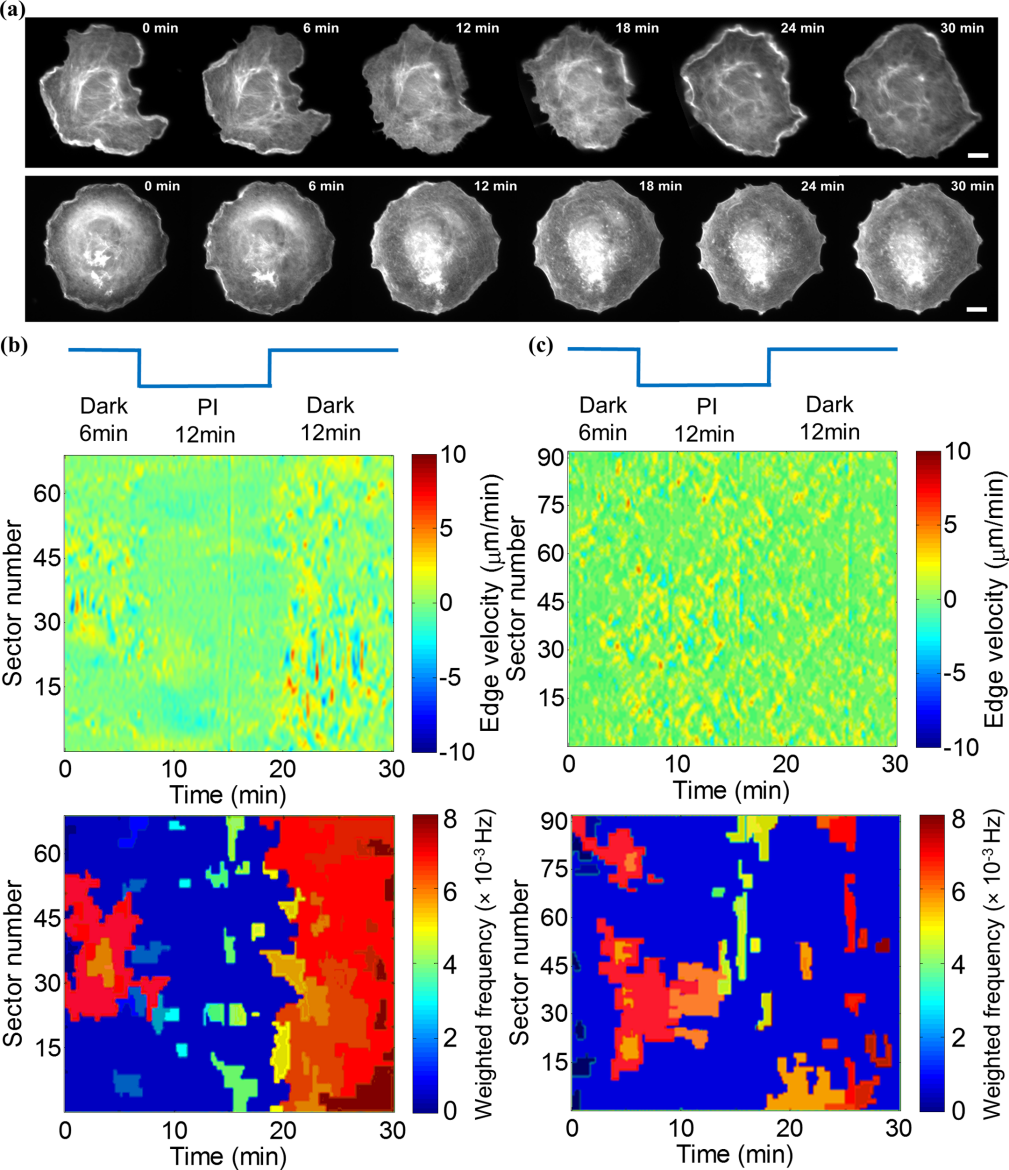
Application of merging algorithm before, during, and after optogenetic Vav2 inhibition in two Cos7 cells with distinct initial morphodynamics. (a) Snapshots of protruding Cos7 cells with strong (top) and weak (bottom) polarity. Scale bar = 25 μm. (b) Top: Protrusion activity map of the Cos7 cell with a strong initial polarity; Bottom: Spectral clustering result (Q = 3). (c) Top: Protrusion activity map of the Cos7 cell with weak polarity; Bottom: Spectral clustering result (Q = 3).

We applied SRM to two cells with distinct initial morphodynamics (Fig 6A, and Videos 6–7). Both cells were perturbed for 12 min by photoinhibition of Vav2 activity and then released for another 12 min. The first cell displayed a clear polarity with a morphodynamically active front between sector 20 and 50 and a more quiescent back. The difference in this activity is easily perceived in the protrusion activity map (Fig 6B, top) and, as with the cell presented in Fig 4, described by clearly separated motion regimens, where the active front breaks into two regimens (red and orange) with slightly different morphodynamic feature values (Fig 6B, bottom). The remainder of the cell edge was described by a single regimen with significantly lower feature values, reflecting the relative quiescence of this cell region. During Vav2 photoinhibition the active front was abrogated and largely merged with the more quiescent regimen. Interestingly, after release from the inhibition the higher activity regimens were restored, yet around the entire cell perimeter. Hence, while the cell regained full morphodynamic activity, it lost polarity. The second cell was less active overall and showed weaker polarity. The effects of Vav2 photo-inhibition were much harder to perceive in the protrusion activity map (Fig 6C, top), yet the region merging unveiled a clear demarcation of motion regimens before and during photo-inhibition (Fig 6C, bottom). After release from inhibition, the cell restored for short time intervals and along the entire perimeter the regimens of the more active zone before inhibition. Together, these experiments highlight the sensitivity of the instantaneous spectral decomposition to outline the spatial and temporal boundaries of distinct morphodynamic activity patterns.

### Motion regimens classified by SRM correspond to spatiotemporal domains of distinct Rac1 signaling

**Fig 7:**
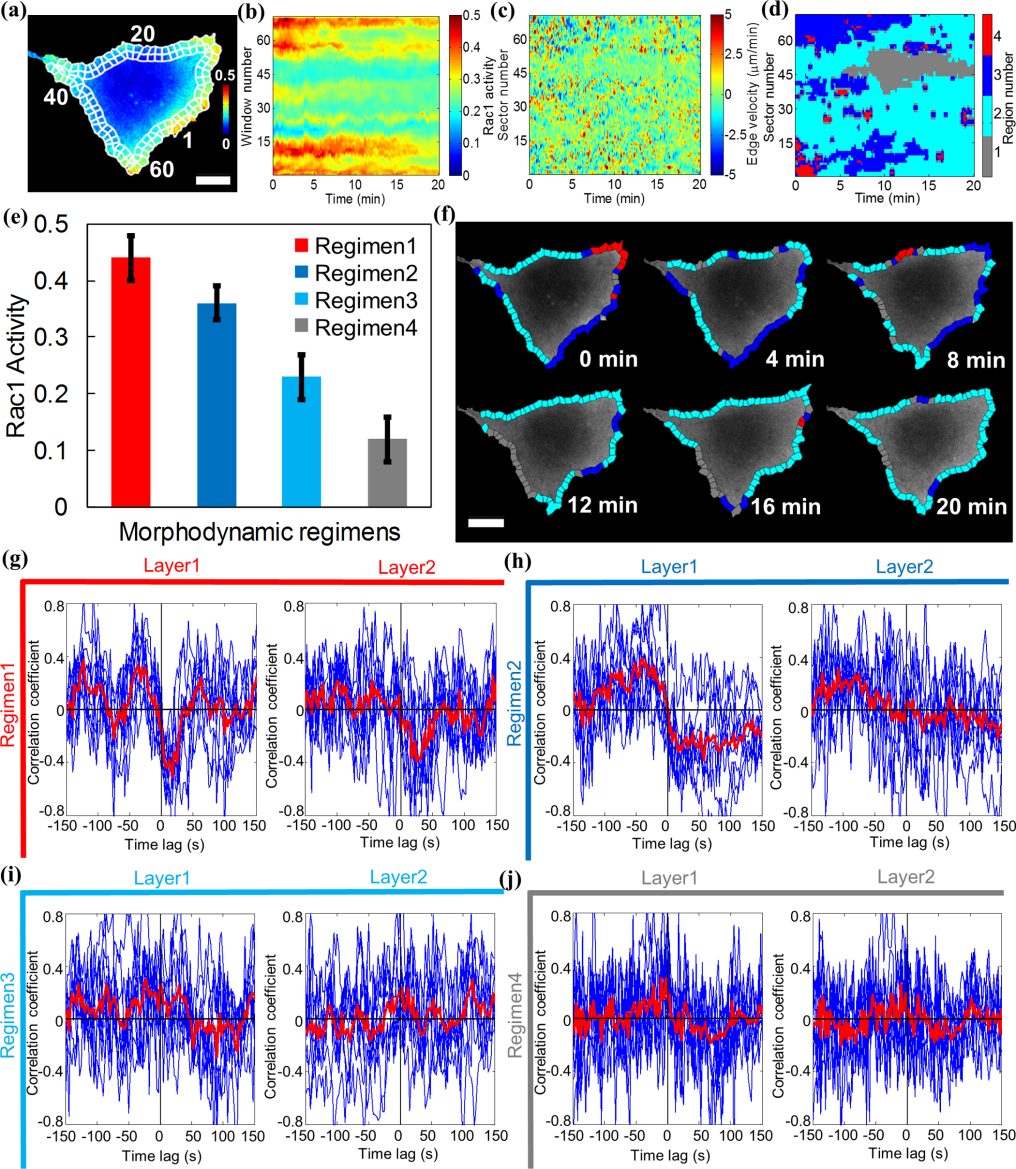
Regions of spectrally homogeneous cell edge motion have distinct Rac1 signaling programs. (a) FRET biosensor probing Rac1 activity (yellow-red tones, high activity; green-blue tones, low activity) in a Cos7 cell. The rectangular boxes with white edges overlaid on cell periphery represent the first (outer) and second (inner) layer of sampling windows. The numbers along the cell periphery mark the locations of sectors/windows. Scale bar = 30 μm. (b) Rac1 activity map of a Cos7 cell. (c) Protrusion activity map of a Cos7 cell. (d) SRM identifying four distinct cell edge motion regimens. (e) Quantification of Rac1 activity in the first layer of probing windows for spectral clustering regimens 1–4. (f) Spectral clustering regions in the first layer of probing windows at six time points of the time lapse sequence (see also Video 9 for the full sequence). Scale bar = 30μm. (g-j) Cross-correlation between edge motion and Rac1 activity in the first and second layer of probing windows for regimens 1–4.

Based on our finding that acute switches in Vav2 activity cause acute shifts in the instantaneous frequency spectra of cell edge motion (Fig 3), we hypothesized that the different motion regimens identified by SRM analysis could be associated with peripheral cell areas of distinct signaling activity. To test this hypothesis, we employed a Förster resonance energy transfer (FRET) biosensor probing the activity of the GTPase Rac1 in Cos7 cells, which is one of the targets of Vav2 (Fig 7A, and Video 8) and a key regulator of cytoskeleton processes implicated in cell protrusion activity. Like the construction of the protrusion activity map, we sampled the Rac1 activity locally in probing windows. Each window corresponded one-to-one with a 3 μm–wide sector for protrusion measurements and had a window depth of 3 μm. The average activity values per probing window for one time point were then pasted into the column of a matrix and the procedure repeated over all time points to generate a *Rac1 activity map* (Fig 7B). Next, we spectrally decomposed the protrusion activity map (Fig 7C) and performed SRM analysis on those spectral features of cell protrusion dynamics to identify distinct motion regimens (Fig 7D). Using Q = 3 we found four distinct regions, each with a different average level of Rac1 activity (Fig 7E). It should be noted that the motion regimens are transient in space and time. We visualized this behavior in a movie where the probing windows are color labeled in correspondence with their association to a particular motion regimen (Video 9; Fig 7F displays selected snapshots at certain time points).

In previous work[45], we demonstrated that cycles of edge protrusion and retraction corresponded to cycles of Rac1 activity. Dependent on the distance from the edge, the motion and signaling cycles had distinct time lags and also showed different levels of correlation, which is a measure of their mutual association. While in the past we manually or semi-manually selected regions along the edge boundary suited for correlation analysis, we wondered whether the boundary regions identified by SRM would now in a more objective manner indicate differences in the magnitude and time lag of the correlation. We performed this region-based correlation analysis in a layer of probing windows at the edge and a second layer of windows shifted into the cell interior by 3 μm, thus covering a band 3–6 μm from the cell edge. Fig 7G–7J display the correlation functions for individual sectors (blue) and their average (red) for the four identified motion regimens in the first and second layers. Both regimens 1 and 2 displayed correlation functions with significant positive lobes for negative time lags and negative lobes for positive time lags in the first layer (Fig 7G and 7H). Consistent with our previous analyses of Rac1 activation in protrusion-retraction cycles[45], this meant that in these regions Rac1 activity was delayed by ~50–60 sec relative to cell protrusion, whereas Rac1 activity was minimal ~30–40 sec prior to protrusion events. Neither regimen 3 nor 4 displayed a significant correlation function in the first or second layer, indicating overall weaker Rac1 signaling in these regions, and especially a weaker coupling between edge motion and Rac1 activity (Fig 7I and 7J). None of the four identified motion regimens displayed significant correlations between Rac1 activity in the second layer and edge motion. This is also consistent with our previous data[45], which showed a decay of spatially finer sampled correlation values to insignificant values at 4.5 μm and longer distances. The correlation functions in the first layer for regimens 1 and 2, however, showed a remarkable difference in the widths of the significant lobes. Regimen 1 had nearly symmetric lobes with a full width at half maximum (FWHM) of 40 sec, whereas regimen 2 had skewed lobes with a FWHM of 75 sec, We note that in previous analyses of correlations between molecular and cell protrusion activities such differences were obscured by the need for averaging over multiple sectors. It is tempting to speculate that the differences in signaling dynamics identified between regimen 1 and 2 are associated with different molecular programs driving Rac1 activity. With the presented SRM analysis of motion regimens, we now have the tool to systematically probe subcellular signaling activities that may even be transient in space and time, and relate them to cell morphogenesis and other cell functions.

## Discussion

In this work we implemented a framework for profiling cellular morphodynamics using spectral decomposition, instantaneous frequency analysis, and unsupervised clustering. First, we extracted the local dynamics of cell edge motion from time-lapse live cell image sequences by sampling protrusion and retraction velocities in discretized sectors of ~3 μm width along the cell periphery. Then, we conducted in every sector HHT-based spectral decomposition of the sampled velocity time series. The HHT resulted in several intrinsic mode functions, here fixed to six, each of which was transformed into instantaneous frequency and amplitude distributions. Hence, unlike a static spectral decomposition such as a Fourier Transform, the HHT-based decomposition captures variations in the oscillatory behavior between different time points and thereby allows detection of switches in the spectrum.

A critical question to address in our development was how much the uncertainty of mapping the displacements of a cell edge between consecutive frames affects the spectral decomposition analysis. Given the previously published mapping algorithm (see Methods), we performed a worst-case error assessment for the displacement data and then simulated how such an error level projects into the distribution of instantaneous frequencies (S5 and S6 Figs). Specifically, our algorithm for computing edge displacements at the level of single pixel guarantees topological consistency among the virtual edge markers between consecutive frames, i.e. protrusion vectors are never allowed to cross each other (S1 Fig). Hence, the maximal mapping error corresponds to the length difference Δ*d*_p_ of a vector that originates from a virtual edge marker at time t and targets one of the neighboring virtual marker positions at time t+1 (S5A and S5B Fig). We then defined a relative mapping error rate (S5B Fig), which for all marker points and an entire movie was approximately uniformly distributed between 0 and 8%, with an average mapping error rate of ~4% (S5C Fig). To assess the effect of such errors on the spectral analysis we randomly perturbed an actual protrusion activity map (S6A Fig) with error rates spanning from 1% to 100% (S6B–S6F Fig) and computed the K-S statistics between the frequency distributions of original and perturbed protrusion time series (S6G Fig). The simulations showed that mapping error rates of less than 10% generate deviations from the ground truth with K-S statistics less than the threshold value ~0.08 associated with the average variation of protrusion dynamics in an molecularly homogeneous cell population (Fig 2F). Hence, we were assured that the 4% level of errors from the cell edge tracking per se does not significantly contribute to conclusions from spectral analysis of morphodynamic behaviors.

Biologically, the most striking finding in this first study with HHT-based protrusion analysis is the distinct, nearly orthogonal meaning of instantaneous frequency and instantaneous amplitude spectra in terms of protrusion regulation. While the amplitude spectra report the speed of cell edge motion, the frequency spectra report how protrusion and retraction cycles are regulated. This is consistent with previous reports from our and other labs that have shown high sensitivity of measurements of protrusion persistence to perturbation of regulatory signals, whereas measurements of protrusion speed were largely unaffected by these same manipulations[44, 46, 58–60]. Cell protrusion requires on the one hand a process that stalls retraction and initiates forward edge motion. On the other hand, it requires a process that stabilizes and eventually reinforces the forward motion against increasing mechanical resistance by the environment and/or the stretched cell plasma membrane[42]. While initiation is stimulated by cell external signals or occurs spontaneously, as is the case for all data analyzed in this work, persistent edge advancement depends on the coordinated engagement of signaling pathways that converge on the activation of a series of nucleators and modulators for actin filament assembly after protrusion onset[61]. Many of these pathways are regulated by feedbacks, which integrate environmental and cell-intrinsic mechanical and chemical cues. Accordingly, dynamic or permanent changes in environmental cues, or in the pathways that process them, primarily affect the protrusion persistence. In contrast, the protrusion speed is less sensitive to the coordination of pathway engagement but more on the overall level of engagement. Moreover, the maximal velocity is reached in the early phases of protrusion, before the pathways critical for reinforcement are engaged[42, 61]. This temporal separation of molecular processes that affect protrusion speed from processes that affect protrusion reinforcement explains also the orthogonality between maximal velocity and persistence measurements. To demonstrate the orthogonality of speed-related amplitude spectra and regulation-related frequency spectra we employed a recently developed optogenetic toolkit to instantaneously deactivate and reactivate a specific node in one of the regulatory pathways. We chose the GEF Vav2, which is one of several activators of the Rac1 GTPase[38, 56] implicated in the assembly of actin filaments required for lamellipodia-driven cell protrusion[62]. Given the redundancy of Vav2 with other Rac1 GEFs, we suspected that inactivation of Vav2 would cause a rewiring of the regulatory circuitry without completely shutting down the regulation process. Indeed, we found a light-dose dependent shift in the frequency spectra. The amplitude spectra, on the other hand, were remarkably stable across the range of applied Vav2 inhibition. This underscores the orthogonality between amplitude and frequency of spontaneous protrusion-retraction cycles, allowing us to distinguish molecular processes implicated in setting the activation level of the protrusion machinery from processes that control protrusion regulation.

HHT-based profiles now introduce a refined framework to describe the state of the regulatory circuitry. Compared to the analysis of protrusion persistence, which requires time integration over multiple cycles, the decomposition of the motion signal into instantaneous frequencies allows distinction of spatially and temporally localized states of the circuitry. We therefore thought that the HHT-based spectra would allow us to define a multidimensional feature vector to distinguish edge sectors with transiently consistent morphodynamic behavior. We also hypothesized that these sectors would correspond to significantly different regulatory signaling activities, i.e. be associated with transient ‘signaling microdomains’[63]. We tested this by separately analyzing both the activation levels of Rac1 as well as the temporal correlation between Rac1 activity and cell edge motion in edge sectors belonging to distinct morphodynamic regimens. The temporal correlation is a surrogate for the coupling of Rac1 signaling with motion, i.e. how much the pathways downstream of Rac1 activation contribute to the morphodynamics analyzed by HHT-based profiling. Indeed, we found that cell edge regions with different morphodynamic behavior displayed different Rac1 activation patterns. This demonstrates that the profiling framework not only detects differences between cells with different molecular makeups, but also provides the means to identify subcellular regions with distinct signaling activities. A critical future application of this capacity will be in the analysis of signal transduction pathways implicated in the regulation of cell shape and migration. In the past, we have manually selected sectors obeying qualitative criteria of cell edge dynamics to perform image fluctuation-based analyses of signaling activities[44, 45, 47], or have included all of the cell edge independent of their dynamics. In both cases, the spatiotemporal averaging necessary to extract meaningful information from signaling fluctuations was executed over the boundaries of signaling microdomains, resulting in less sensitivity and bias. Moving forward, HHT-based morphodynamic profiling and spatiotemporal clustering of similar profiles will be used to automatically identify sub-cellular regions of homogeneous signaling activities with high resolution in both time and space. A second future application of HHT-based frequency decomposition and spatiotemporal clustering will be in the time series analysis of activity biosensor fluctuations per se. While the presented work focused on domain definitions only of motion fluctuations, the same framework could also be applied to fluctuations in signal activation throughout the entire cells. This will potentially enable the complete mapping of sub-cellular signaling regimes, and in combination with perturbations of signaling nodes, the identification of sub-cellularly distributed functions of signals, which is currently experimentally inaccessible.

In sum, HHT-based profiling and clustering will have numerous powerful applications in the quantitative analysis of cell behavior, from classifying whole-cell migration states to focusing on subcellular regulatory microdomains. The software to enable these types of analyses is accessible under the Github link: https://github.com/DanuserLab/MorphodynamicProfiling and https://github.com/DanuserLab/Windowing-Protrusion.

## Methods

### Cell segmentation

To identify the cell-edge location in the examples presented here, automatic thresholding was combined with morphological post-processing. Thresholds were automatically selected by fitting a smoothing spline to the image intensity histogram and by finding the first local minimum after the lowest-intensity maximum, thus selecting a threshold, which separates the low-intensity histogram mode corresponding to the background from the higher-intensity peak(s) associated with cellular fluorescence. In cases where this automatic approach failed, thresholds were manually selected. Images were pre-filtered with a Gaussian approximation of the point spread function prior to binarization by thresholding to minimize the effects of image noise. To ensure that the resulting segmentation contained only a single connected component corresponding to the cell, the thresholding was followed by automated morphological post-processing including hole-filling for small intracellular areas of low intensity, a closure operation to fill small gaps in low-intensity edge regions, and only the largest remaining connected component was retained to remove small background spots.

### Cell-edge velocity calculation

Cell edge velocities were derived from pixel-by-pixel matching of cell contours between consecutive time points, as described in ref. [64] and reproduced here for completeness. In summary, a B-form spline was fitted to the edge pixel positions of the segmented cell area, with nodes corresponding to each edge pixel (S1A Fig). The spline representations of two consecutive frames were then divided into segments between their intersections. To map a correspondence between the edge splines on consecutive frames, the following objective function was iteratively minimized:

$$\left({\hat{o}}_{1},\ldots ,{\hat{o}}_{n}\right)=\underset{\left({\hat{o}}_{1},\ldots ,{\hat{o}}_{n}\right)}{\text{arg}\;\text{min}}\{\begin{array}{cc} \sum_{i=1}^{n}{\left[x(t+1,o_{i})-x(t,p_{i})\right]}^{2}+ & \omega \sum_{i=2}^{n}{\left[\frac{o_{i}(t+1)-o_{i-1}(t+1)}{p_{i}(t)-p_{i-1}(t)}\right]}^{2}\\ {SUM}_{A} & {SUM}_{B} \end{array}\}$$ (M.1)

$$\text{with}\;\text{the}\;\text{topological}\;\text{constraints}\;e_{1}=o_{1}<o_{2}<\ldots <o_{n}=e_{n}$$ (M.2)

The variable *n* denotes the number of nodes, which in the absence of down-sampling (see below) is equal to the number of edge pixels in that segment. $p_{1,2,\ldots n}\left(t\right)$ are the parameters of the spline at time *t* defining equally spaced edge nodes $x(t,p_{i})$, one at each edge pixel. The goal of Eqs M.1 and M.2 is to identify *n* spline parameters $o_{1,2,\ldots n}\left(t+1\right)$ in between the intersection points $e_{1}\;$and $e_{n}\;$that define non-equally spaced nodes $x(t+1,o_{i})$ at *t+1* such that the overall displacement (${SUM}_{A}$) and strain, i.e. changes in spacing of nodes (${SUM}_{B}$) is minimized. M.2 imposes the constraint to the minimization that displacement vectors must not cross. The two sums in M.1 have different physical units. To balance them correctly we introduce a factor *ω* as follows:

$$\omega =w*{\left(\frac{SUM_{A}}{SUM_{B}}\right)}_{iteration=1}=w*{\left\{\frac{\sum_{i=1}^{n}{\left[x\left(t+1,o_{i}\right)-x\left(t,p_{i}\right)\right]}^{2}}{\sum_{i=2}^{n}{\left[\frac{o_{i}\left(t+1\right)-o_{i-1}\left(t+1\right)}{p_{i}(t)-p_{i-1}(t)}\right]}^{2}}\right\}}_{iteration=1}$$ (M.3)

The factor *ω* is calculated only in the first iteration of the minimization, as the unit conversion by the ratio ${SUM}_{A}/{SUM}_{B}$ changes insubstantially thereafter. The parameter *w* is a free user-control that allows the definition of the trade-off between minimal edge displacement and minimal lateral strain (S1B–S1E Fig). For *w* = 1 these two competing criteria have equal weight. The global solution of the edge mapping is fairly insensitive to the value of *w*. However, adjustments may be useful to track particularly rugged features of the cell edge, or vice versa, to oppress the mapping of spiky edge features.

The minimization of Eq M.1 can be computationally costly when the number of edge pixels in a segment exceeds 100. To circumvent this problem, we introduce a control parameter $10<N_{max}<100$. When the number of edge pixels in a segment is greater than $N_{max}$, we downsample the number of nodes to $N_{max}$, calculate the boundary displacement for this number of nodes, and then up-sample to the original number of edge pixels by interpolation. This control parameter therefore not only allows the flexibility of trading computational speed for accuracy, but allows the method to be applied to cells of any size imaged at any resolution. Once the boundary displacements are identified, the projections of these displacements onto the boundary normal vector are calculated to obtain a signed local measurement of the instantaneous normal edge velocity. The nodes are reset with every time step (S1F Fig). Accordingly, to compute a continuous path for a virtual edge marker throughout an entire movie it is necessary to interpolate marker positions for each time point (not applied in this study).0020

### Sampling window generation for the readout of Rac1 fluctuations

Our software supports two methods for sampling window creation. The first is a discrete pixel space method, which is faster and ensures windowing of the entire segmented area. The second is a sub-pixel method, which allows more flexibility and precision, but which excludes some segmented areas that do not meet strict criteria. The second method is also more computationally intensive. In both methods the intracellular frame of reference used to create the image sampling windows is based on the Euclidean distance transform *D* of the cell edge[65].

The discrete pixel space sampling window generation method creates sampling windows using both the discrete distance transform *D* and the nearest-neighbor transform or feature transform *F*:

$$d_{i}=D\left(u_{i},x_{1,2,\ldots n}\right)$$ (M.4)

$$f_{i}=F\left(u_{i},x_{1,2,\ldots n}\right)$$ (M.5)

where, for the *i*th pixel ***u***_*i*_ in the segmented cell, $f_{i}$ is the index of the closest pixel on the cell boundary ***x***, and *d*_*i*_ is the distance to this pixel and therefore the shortest distance to the cell boundary. We then also calculate the associated distance *along* the cell boundary for each pixel,

$$l_{i}=L\left(u_{i}\right)=\overset{f_{i}}{\underset{k=2}{\sum }}\left|x_{k}-x_{k-1}\right|$$ (M.6)

The location of the origin of the sampling windows, ***x***_*1*_, is determined by the user. A given sampling window can then be defined as:

$$W_{m,p}=\left\{u_{1,2,\ldots ,I}\text{|}u_{i}\in \Omega \wedge \;b_{m}<d_{i}\leq b_{m+1}\wedge \;s_{p}<l_{i}\leq s_{p+1}\right\}$$ (M.7)

where Ω is the segmented cell area, *b*_*1*,*2*,*…M*_ are the user-selected distances from the cell edge, and *s*_*1*,*2*,*…P*_ are the user-selected distances along the cell edge. That is, a particular window *W*_*m*,*p*_ is defined as all pixels with distances between *b*_*m*_ and *b*_*m+1*_ from the cell edge, and for which the distance from the origin along the closest cell edge is between *s*_*p*_ and *s*_*p+1*_. Note that in discrete pixel space it is non-trivial to define a distance measure *L* at a contour other than the cell boundary ***x***. This is because with near convex cell edges the nearest feature transform, $f_{i}$ at positions inwards from the cell edge will not contain indexing which represents all of the pixels on the cell boundary. Therefore, the discrete windowing approach in our software package does not currently support specification of a ‘master contour’ other than the cell edge.

In the sub-pixel windowing method, the cell interior is subdivided with respect to distance from the edge by defining isocontours (or level sets) *C* of the Euclidean distance transform *D* at distance isovalues specified by the user:

$$C_{m}=\left\{u_{1},‥,u_{n}\text{|}D\left(u_{1},‥,u_{n}\right)=b_{m}\right\}$$ (M.8)

Where *C*_*m*_ is the *m*^th^ isocontour at the distance value *b*_*m*_. Isocontour coordinates are refined to sub-pixel precision by linear interpolation of the original distance transform, which is calculated on the discrete pixel grid. The subdivisions of the cell interior into window slices are defined by first defining ‘slice’ start positions ***σ*** along a user-specified ‘master-contour’ *C*_*μ*_:

$$\sigma =\left\{C_{\mu ,1},‥,C_{\mu ,n}\text{|}L_{\mu }\left(C_{\mu }\right)\in s_{1,2,\ldots P}\right\}$$ (M.9)

Where *s*_*1*,*2*,*…P*_ are again the user-selected distances along the master contour *μ* and

$$L_{\mu }\left(C_{\mu ,i}\right)=\overset{i}{\underset{k=2}{\sum }}\left|C_{\mu ,k}-C_{\mu ,k-1}\right|$$ (M.10)

is the distance along the master contour from the origin *C*_*μ*,*1*_. The position of this origin can be set by the user as well, e.g. to mark the back or front of the cell. Note that this choice has no influence on the actual geometry of the sampling windows. The ‘slice’ curves *S* used to subdivide the cell from these slice start positions are then determined by a maximal-gradient ascent of the Euclidean distance transform:

$$S_{p,i}=S_{p,i-1}+\nabla D\left(S_{p,i-1}\right)$$ (M.11)

with

$$S_{p,1}=\sigma_{p}$$ (M.12)

and the local gradients are again estimated via linear interpolation. The geometry of an individual sampling window *W*_*m*,*p*_ is then defined as the area enclosed by the two isocontours *C*_*m*_ and *C*_*m+1*_ and the slice curves *S*_*p*_ and *S*_*p+1*_. This ensures that the image area within each sampling window occupies a specific range of distances from the cell edge, and that the closest region of the cell edge is the one delineated by the intersection of gradient ascent polygons and the cell edge. Regions of the cell interior that do not meet these criteria are excluded from the windowing. This includes regions spanning ridges in the distance transform, which are therefore equally proximal to two disconnected regions of the cell edge, and regions near image borders, where the association with the cell edge is indeterminate.

### Sampling window propagation

In an image time-lapse sequence, the position of the sampling windows in each frame can be determined in several ways: The algorithm described above can be applied to each frame using constant isocontour and gradient ascent curve spacing, and only the location of the origin varies with time. The location of this origin is propagated between subsequent frames either by using the closest edge displacement vector or by finding the closest point on each subsequent cell edge to the original user-selected origin. In either of these cases the number of sampling window slices can vary with respect to time if the length of the cell edge changes. Alternatively, the number of sampling window slices can be held constant, allowing the width of each to vary as the length of the cell edge changes. Finally, it is also possible to allow each gradient ascent start-point to follow the edge-displacement vectors adjacent to it. This propagation method will maintain the number of window sampling slices, but will allow each slice to expand or contract as the associated region of the cell edge protrudes or retracts. This last method tends to generate the most stable window configurations. Irrespective of the propagation method chosen, each sampling window band will always maintain its distance from the cell edge. For all analyses in Fig 7 we used the setting with constant number of window sampling slices.

### Image and edge velocity sampling

Once the sampling windows are generated for each image of a dataset, the associated image signals, image-derived data and edge velocities can be sampled. For image sampling, a variety of statistics are calculated (mean, standard deviation, maximum etc.) for each pixel whose center lies within a given sampling window, yielding sample statistics in the activity matrix for each sampling window at each time point. For sampling of edge velocities, statistics are calculated for the displacement vectors associated with a particular cell edge sector. For example, instantaneous cell edge velocities are calculated for each edge sector as the average of the projections of the displacement vectors onto the associated edge normal divided by the time interval between frames. Because the sample sizes per edge sector may vary with the local cell edge geometry and motion, the number of edge displacement vectors contributing to each sample is also quantified.

### Live cell imaging

Cos7 cells were maintained in DMEM growth medium supplemented with 10% (vol/vol) FBS at 37°C and 10% CO2. The transient transfection of Cos7 cells were performed using Fugene 6 transfection reagent (Promega) under the guidelines of the manufacturer. The YFP-PI(WT)-Vav2(DPZ) (Addgene #86974) and mCherry-lifeAct expressing plasmids were used to photo-inhibit Vav2 and label actin[38]. For 48 spontaneously migrating cells, a plasmid expressing mCherry-lifeAct was used. To monitor Rac1 activity, cells were transfected with dual chain Rac1 biosensor Rac1FLARE.dc1g[45] that has a dTurquoise and YPet fluorescent protein pair. To obtain a fixed ratio of two chains, two consecutive 2A viral peptide sequences from porcine teschovirus-1 (P2A) and Thosea asigna virus (T2A) were inserted between two chains, leading to cleavage of the two chains during translation.

For live cell imaging, cells were plated on sterilized coverslips coated with 5 μg/mL of fibronectin (Sigma) and incubated in DMEM growth medium supplemented with 10% (vol/vol) FBS at 37°C. On the day of imaging, cell medium was replaced with L15 imaging medium (Invitrogen) supplemented with 5% (vol/vol) FBS. The coverslips with cells were placed in an open heated chamber (Warner Instruments) and live cell imaging was performed with an Olympus IX-81 inverted epifluorescence microscope equipped with an Olympus 40x UPlan FLN1.25 N/A silicon oil objective and a Flash 4 sCMOS camera (Hamamatsu) with temperature control (BC-100 20/20 Technology). For excitation, a 100 Watt mercury arc lamp with a 3% ND filter and a 510–520 (YFP) nm or 565–595 (mCherry) nm band-pass filter was employed. A 1% ND filter and a 426–446 nm band-pass filter were used with a 100 Watt mercury arc lamp (~ 1 nW/μm^2^ of power density at λ = 488 nm, measured at the specimen plane) for blue light pulse illumination. For emission ratio imaging of Rac1, CFP: (ex)FF-434/17, (em)FF-482/35; FRET: (ex)FF-434/17, (em)FF-550/49; YFP: (ex) FF-510/10, (em)FF-550/49 filters (Semrock) were used. Images of control cells and cells expressing the PI-Vav2, and cells expressing Rac1 biosensor were taken every 10 and 5 sec, respectively.

## Supporting information

S1 Fig Edge displacement calculation. (a) Displacement vectors (blue arrows) connecting the cell edges between consecutive frames (red and green lines). These are calculated independently for each cell edge segment in between intersections of the edges (purple circles) by minimizing the sum of total displacement magnitude and lateral strain generated by the mapping. (b-d) Displacement vectors calculated by minimizing the total displacement magnitude only (*w* = 0; b), strain only (*w* >> 1; c), or the sum of equally weighted displacement magnitude and strain (*w* = 1; d). (e) Normalized mean-square-error (MSE) in edge velocities computed for a range of possible values for the control parameters *w* (red line), and for the same data down-sampled in time by a factor of 10 (blue line). The reference for the error calculation is defined by the edge velocities computed for *w* = 1 at full time resolution. (f) Displacement vectors (blue) are calculated for all pairs of consecutive edges (black), connecting the cell edge in early time points (lighter colors) to later time points (darker colors). Scale bars: 5μm (a), 5 pixels (b-d).
(DOCX)

S2 Fig Selected snapshots of cell edge configurations and protrusion activity maps for the six intrinsic mode functions (IMFs) retrieved after empirical mode decomposition of the edge motion of a cell with strong polarization and significant protrusion activity. (a-f) Upper panels of three snapshots: simulated cell edge images at t = 0, 15 and 30 min for each IMF. Lower panel: protrusion activity maps for each IMF. More detailed cell shape propagation over time is shown in Video 2.
(DOCX)

S3 Fig Cumulative distribution function (CDF) comparison of instantaneous frequency distributions for all intrinsic mode functions (IMFs) between an active and a quiescent Cos7 cell. P-value is calculated by Kolmogorov–Smirnov (K-S) test. From (a) to (f), results of IMF1 till IMF6 are presented. Left: CDFs of instantaneous frequency; Right: CDFs of instantaneous amplitude.
(DOCX)

S4 Fig Comparison of instantaneous frequency distributions for all intrinsic mode functions (IMFs) collected before and during a PI period composed of 1000 msec pulses of light interspersed with 9000 msec darkness, 100 msec pulses of light interspersed with 9900 msec darkness, and 1 msec pulses of light interspersed with 9999 msec darkness. From (a) to (f), results of IMF1 till IMF6 are presented. Left: pulse length of 1000 msec; Middle: pulse length of 100 msec; Right: pulse length of 1 msec. P-value is calculated by K-S test.
(DOCX)

S5 Fig Statistic analysis on lateral shift error for mapping consecutive cell edge outlines. (a) Left: the overlaid consecutive cell edge outlines at t (blue) and t+1 (red). Right: the zoom-in portion of the localized protrusion regions. The grey solid arrows representing the protrusion vectors that map the two consecutive outlines. One of them colored in black is taken as an example, two possible inaccurate mapping vectors are shown in dash black arrows, and the associated lateral shift error vectors are presented in solid green arrows. (b) Schematic illustration of mapping error rate computation. (c) Histogram of mapping error rate over all pixels on cell edge and whole time frames.
(DOCX)

S6 Fig Analysis of the possible influence of edge mapping errors. (a) Original protrusion activity map. (b-f) Protrusion activity maps with random mapping errors superimposed at rate levels 1%, 3%, 10%, 30% to 100%. See S5 Fig for a definition of the error rate. (g) K-S statistics comparing the instantaneous frequency spectra distributions for IMF1 and IMF2 between the original protrusion activity map and error-perturbed maps. The dashed line referenced the threshold K-S statistics derived from the average of K-S statistics between cell pairs in a population with similar molecular make-up (average of heatmap Fig 2F).
(DOCX)

S1 Video Cos7 cell migrating with persistent polarity and protrusion/retraction over large parts of its periphery. Overlay, computationally segmented cell edges color-coded from early time points, blue, to late time points, red. Movie duration: 30 min; scale bar: 20 μm.
(AVI)

S2 Video Simulation of time-lapse sequences of cell edge motion captured by intrinsic mode functions (IMFs) 1–6. The simulation is applied to the outline of the Cos7 cell shown in Video 1. Movie duration: 30 min; scale bar: 20 μm.
(AVI)

S3 Video Quiescent Cos7 cell with unpolarized morphology and small oscillatory edge movements. Overlay, computationally segmented cell edges color-coded from early time points, blue, to late time points, red. Movie duration: 30 min; scale bar: 20 μm.
(AVI)

S4 Video Active Cos 7 cell migrating with persistent polarity labeled by higher/lower motility subcellular regions (red/blue) over time. Movie duration: 30 min; scale bar: 20 μm.
(AVI)

S5 Video Quiescent Cos 7 cell with unpolarized morphology labeled by higher/lower motility subcellular regions (red/blue) over time. Movie duration: 30 min; scale bar: 20 μm.
(AVI)

S6 Video Cos7 cell under photo-inhibition of Vav2 from 6 till 18 min. Before inhibition, the cell shows polarity. After reactivation of Vav2 beyond 18 min, the polarity is eliminated and replaced by protrusion activity along the entire periphery. Movie duration: 30 min; scale bar: 25 μm.
(AVI)

S7 Video Cos7 cell under photo-inhibited Vav2 from 6 till 18 min. Before inhibition, the cell shows weak polarity. After reactivation of Vav2 beyond 18 min, the cell shows unpolarized bursts of protrusion along the entire periphery. Movie duration: 30 min; scale bar: 25 μm.
(AVI)

S8 Video Cos7 cell expressing FRET biosensor of Rac1signaling activity. Image signal shows normalized FRET intensity indicating Rac1 activity level. Movie duration: 20 min; scale bar: 30 μm.
(AVI)

S9 Video Cell shown in Video 8 overlaid by color-coded edge sectors indicating motion regimens as classified by statistical region merging of the protrusion activity map in Fig 7C. Movie duration: 20 min; scale bar: 30 μm.
(AVI)
