# Supplementary material for: Profiling cellular morphodynamics by spatiotemporal spectrum decomposition
Source: PLoS Comput Biol. 2018 Aug 2;14(8):e1006321. doi: 10.1371/journal.pcbi.1006321 (PMC6091976; doi:10.1371/journal.pcbi.1006321)
Supplement: S1 Fig — (a) Displacement vectors (blue arrows) connecting the cell edges between consecutive frames (red and green lines). These are calculated independently for each cell edge segment in between intersections of the edges (purple circles) by minimizing the sum of total displacement magnitude and lateral strain generated by the mapping. (b-d) Displacement vectors calculated by minimizing the total displacement magnitude only (w = 0; b), strain only (w >> 1; c), or the sum of equally weighted displacement magnitude and strain (w = 1; d). (e) Normalized mean-square-error (MSE) in edge velocities computed for a range of possible values for the control parameters w (red line), and for the same data down-sampled in time by a factor of 10 (blue line). The reference for the error calculation is defined by the edge velocities computed for w = 1 at full time resolution. (f) Displacement vectors (blue) are calculated for all pairs of consecutive edges (black), connecting the cell edge in early time points (lighter colors) to later time points (darker colors). Scale bars: 5μm (a), 5 pixels (b-d). (DOCX) [file pcbi.1006321.s001.docx]

S1 Fig Edge displacement calculation. (a) Displacement vectors (blue arrows) connecting the cell edges between consecutive frames (red and green lines). These are calculated independently for each cell edge segment in between intersections of the edges (purple circles) by minimizing the sum of total displacement magnitude and lateral strain generated by the mapping. (b-d) Displacement vectors calculated by minimizing the total displacement magnitude only (*w* = 0; b), strain only (*w* >> 1; c), or the sum of equally weighted displacement magnitude and strain (*w* = 1; d). (e) Normalized mean-square-error (MSE) in edge velocities computed for a range of possible values for the control parameters $\boldsymbol{w}$ (red line), and for the same data down-sampled in time by a factor of 10 (blue line). The reference for the error calculation is defined by the edge velocities computed for *w* = 1 at full time resolution. (f) Displacement vectors (blue) are calculated for all pairs of consecutive edges (black), connecting the cell edge in early time points (lighter colors) to later time points (darker colors). Scale bars: 5μm (a), 5 pixels (b-d).
