# Supplementary material for: Profiling cellular morphodynamics by spatiotemporal spectrum decomposition
Source: PLoS Comput Biol. 2018 Aug 2;14(8):e1006321. doi: 10.1371/journal.pcbi.1006321 (PMC6091976; doi:10.1371/journal.pcbi.1006321)
Supplement: S2 Fig — (a-f) Upper panels of three snapshots: simulated cell edge images at t = 0, 15 and 30 min for each IMF. Lower panel: protrusion activity maps for each IMF. More detailed cell shape propagation over time is shown in Video 2. (DOCX) [file pcbi.1006321.s002.docx]

**S2 Fig** Selected snapshots of cell edge configurations and protrusion activity maps for the six intrinsic mode functions (IMFs) retrieved after empirical mode decomposition of the edge motion of a cell with strong polarization and significant protrusion activity. (a-f) Upper panels of three snapshots: simulated cell edge images at t = 0, 15 and 30 min for each IMF. Lower panel: protrusion activity maps for each IMF. More detailed cell shape propagation over time is shown in Video 2.
