# Supplementary material for: Profiling cellular morphodynamics by spatiotemporal spectrum decomposition
Source: PLoS Comput Biol. 2018 Aug 2;14(8):e1006321. doi: 10.1371/journal.pcbi.1006321 (PMC6091976; doi:10.1371/journal.pcbi.1006321)
Supplement: S3 Fig — P-value is calculated by Kolmogorov–Smirnov (K-S) test. From (a) to (f), results of IMF1 till IMF6 are presented. Left: CDFs of instantaneous frequency; Right: CDFs of instantaneous amplitude. (DOCX) [file pcbi.1006321.s003.docx]

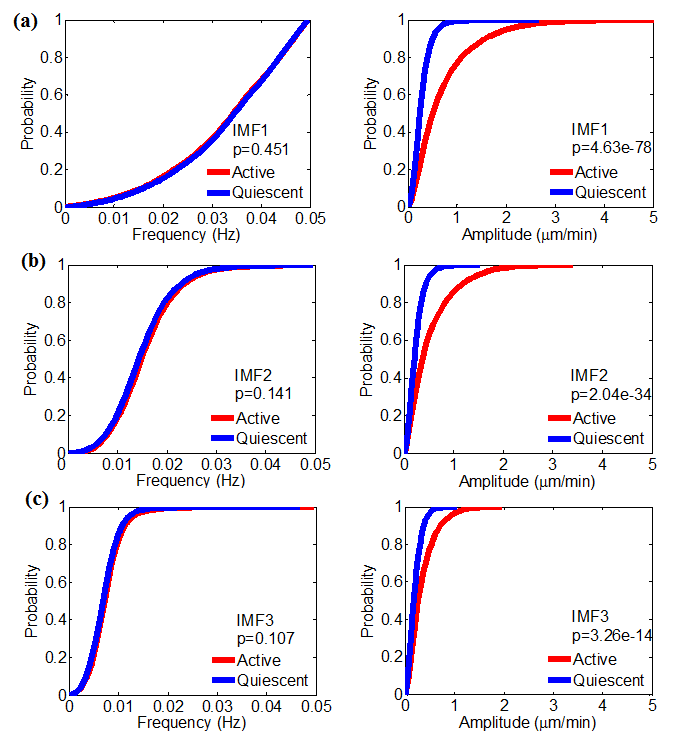


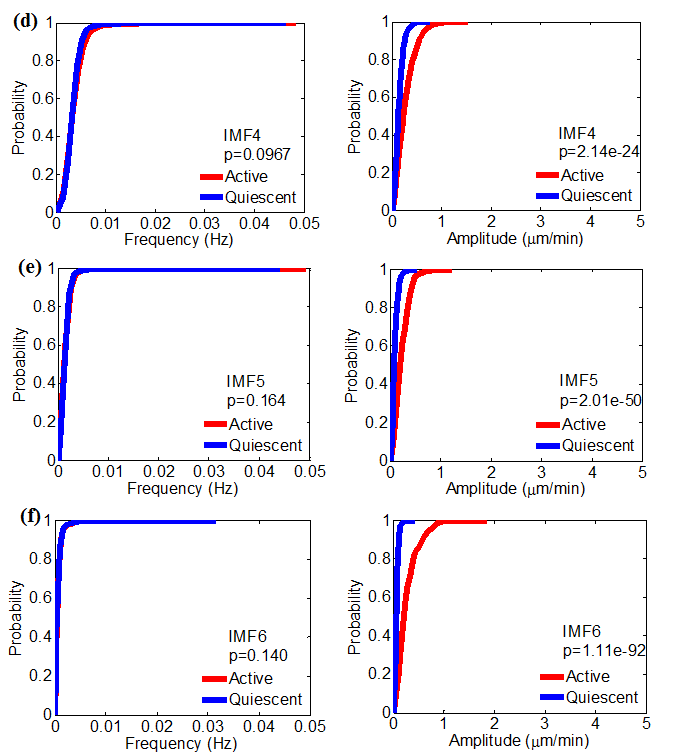
**S3 Fig** Cumulative distribution function (CDF) comparison of instantaneous frequency distributions for all intrinsic mode functions (IMFs) between an active and a quiescent Cos7 cell. P-value is calculated by Kolmogorov–Smirnov (K-S) test. From (a) to (f), results of IMF1 till IMF6 are presented. Left: CDFs of instantaneous frequency; Right: CDFs of instantaneous amplitude.
