# Supplementary material for: Profiling cellular morphodynamics by spatiotemporal spectrum decomposition
Source: PLoS Comput Biol. 2018 Aug 2;14(8):e1006321. doi: 10.1371/journal.pcbi.1006321 (PMC6091976; doi:10.1371/journal.pcbi.1006321)
Supplement: S4 Fig — From (a) to (f), results of IMF1 till IMF6 are presented. Left: pulse length of 1000 msec; Middle: pulse length of 100 msec; Right: pulse length of 1 msec. P-value is calculated by K-S test. (DOCX) [file pcbi.1006321.s004.docx]

**S4 Fig** Comparison of instantaneous frequency distributions for all intrinsic mode functions (IMFs) collected before and during a PI period composed of 1000 msec pulses of light interspersed with 9000 msec darkness, 100 msec pulses of light interspersed with 9900 msec darkness, and 1 msec pulses of light interspersed with 9999 msec darkness. From (a) to (f), results of IMF1 till IMF6 are presented. Left: pulse length of 1000 msec; Middle: pulse length of 100 msec; Right: pulse length of 1 msec. P-value is calculated by K-S test.
