# Supplementary material for: Profiling cellular morphodynamics by spatiotemporal spectrum decomposition
Source: PLoS Comput Biol. 2018 Aug 2;14(8):e1006321. doi: 10.1371/journal.pcbi.1006321 (PMC6091976; doi:10.1371/journal.pcbi.1006321)
Supplement: S5 Fig — (a) Left: the overlaid consecutive cell edge outlines at t (blue) and t+1 (red). Right: the zoom-in portion of the localized protrusion regions. The grey solid arrows representing the protrusion vectors that map the two consecutive outlines. One of them colored in black is taken as an example, two possible inaccurate mapping vectors are shown in dash black arrows, and the associated lateral shift error vectors are presented in solid green arrows. (b) Schematic illustration of mapping error rate computation. (c) Histogram of mapping error rate over all pixels on cell edge and whole time frames. (DOCX) [file pcbi.1006321.s005.docx]

**S5 Fig** Statistic analysis on lateral shift error for mapping consecutive cell edge outlines. (a) Left: the overlaid consecutive cell edge outlines at t (blue) and t+1 (red). Right: the zoom-in portion of the localized protrusion regions. The grey solid arrows representing the protrusion vectors that map the two consecutive outlines. One of them colored in black is taken as an example, two possible inaccurate mapping vectors are shown in dash black arrows, and the associated lateral shift error vectors are presented in solid green arrows. (b) Schematic illustration of mapping error rate computation. (c) Histogram of mapping error rate over all pixels on cell edge and whole time frames.
