# Supplementary material for: Profiling cellular morphodynamics by spatiotemporal spectrum decomposition
Source: PLoS Comput Biol. 2018 Aug 2;14(8):e1006321. doi: 10.1371/journal.pcbi.1006321 (PMC6091976; doi:10.1371/journal.pcbi.1006321)
Supplement: S6 Fig — (a) Original protrusion activity map. (b-f) Protrusion activity maps with random mapping errors superimposed at rate levels 1%, 3%, 10%, 30% to 100%. See S5 Fig for a definition of the error rate. (g) K-S statistics comparing the instantaneous frequency spectra distributions for IMF1 and IMF2 between the original protrusion activity map and error-perturbed maps. The dashed line referenced the threshold K-S statistics derived from the average of K-S statistics between cell pairs in a population with similar molecular make-up (average of heatmap Fig 2F). (DOCX) [file pcbi.1006321.s006.docx]

**S6 Fig** Analysis of the possible influence of edge mapping errors. (a) Original protrusion activity map. (b-f) Protrusion activity maps with random mapping errors superimposed at rate levels 1%, 3%, 10%, 30% to 100%. See S5 Fig for a definition of the error rate. (g) K-S statistics comparing the instantaneous frequency spectra distributions for IMF1 and IMF2 between the original protrusion activity map and error-perturbed maps. The dashed line referenced the threshold K-S statistics derived from the average of K-S statistics between cell pairs in a population with similar molecular make-up (average of heatmap Fig. 2(f)).
